# Methodological framework for radiomics applications in Hodgkin’s lymphoma

**DOI:** 10.1186/s41824-020-00078-8

**Published:** 2020-06-01

**Authors:** Martina Sollini, Margarita Kirienko, Lara Cavinato, Francesca Ricci, Matteo Biroli, Francesca Ieva, Letizia Calderoni, Elena Tabacchi, Cristina Nanni, Pier Luigi Zinzani, Stefano Fanti, Anna Guidetti, Alessandra Alessi, Paolo Corradini, Ettore Seregni, Carmelo Carlo-Stella, Arturo Chiti

**Affiliations:** 1 Humanitas University, Via Rita Levi Montalcini 4, MI 20090 Pieve Emanuele, Italy; 2 Humanitas Clinical and Research Center – IRCCS -, via Manzoni 56, 20089 Rozzano, MI Italy; 3 MOX–Modelling and Scientific Computing lab., Department of Mathematics, Politecnico di Milano, Milano, Italy; 4 CADS–Center for Analysis, Decision, and Society, Human Technopole, Milano, Italy; 5 Nuclear Medicine, AOU S.Orsola-Malpighi, Bologna, Italy; 6 Institute of Hematology “Seràgnoli”, University of Bologna, Bologna, Italy; 7 Fondazione IRCCS Istituto Nazionale dei Tumori, Milan, Italy; 8 University of Milan, Milan, Italy

**Keywords:** Lymphoma, PET/CT, Radiomics, Similarity, Feature selection, Silhouette, Response prediction, Outcome prediction

## Abstract

**Background:**

According to published data, radiomics features differ between lesions of refractory/relapsing HL patients from those of long-term responders. However, several methodological aspects have not been elucidated yet.

**Purpose:**

The study aimed at setting up a methodological framework in radiomics applications in Hodgkin’s lymphoma (HL), especially at (a) developing a novel feature selection approach, (b) evaluating radiomic intra-patient lesions’ similarity, and (c) classifying relapsing refractory (R/R) vs non-(R/R) patients.

**Methods:**

We retrospectively included 85 patients (male:female = 52:33; median age 35 years, range 19–74). LIFEx (www.lifexsoft.org) was used for [^18^F]FDG-PET/CT segmentation and feature extraction. Features were *a-priori* selected if they were highly correlated or uncorrelated to the volume. Principal component analysis-transformed features were used to build the fingerprints that were tested to assess lesions’ similarity, using the *silhouette*. For intra-patient similarity analysis, we used patients having multiple lesions only. To classify patients as non-R/R and R/R, the fingerprint considering one single lesion (fingerprint_One) and all lesions (fingerprint_All) was tested using Random Undersampling Boosting of Tree Ensemble (RUBTE).

**Results:**

HL fingerprints included up to 15 features. Intra-patient lesion similarity analysis resulted in mean/median silhouette values below 0.5 (low similarity especially in the non-R/R group). In the test set, the fingerprint_One classification accuracy was 62% (78% sensitivity and 53% specificity); the classification by RUBTE using fingerprint_All resulted in 82% accuracy (70% sensitivity and 88% specificity).

**Conclusions:**

Lesion similarity analysis was developed, and it allowed to demonstrate that HL lesions were not homogeneous within patients in terms of radiomics signature. Therefore, a random target lesion selection should not be adopted for radiomics applications. Moreover, the classifier to predict R/R vs non-R/R performed the best when all the lesions were used.

## Introduction

Hodgkin’s lymphoma (HL) is a hematological disease characterized by an excellent long-term outcome (Mottok & Steidl, 2018). However, up to 15% of patients with early stage, and up to 30% of patients with advanced stage HL, are primary refractory or experience recurrence (LaCasce, 2019). Therefore, the identification of cases at high risk for first-line therapy failure or recurrence would significantly impact on HL patient management. Presently, prognostic stratification and, consequently, the therapeutic strategy in HL rely mainly on stage and the presence of risk factors (Ansell, 2018). However, current staging system and prognostic factors provide limited information about the lymphoma biology and fail in identification of refractory HL patients at baseline (Mottok & Steidl, 2018).

Novel strategies for the characterization of disease are emerging. Detection of tumor-specific mutations in cell-free circulating tumor DNA (ctDNA) by next-generation sequencing (NGS) techniques has been described with encouraging results for therapy monitoring and assessment of minimal residual disease (Mottok & Steidl, 2018; Spina et al., 2018). Recently, radiomics and artificial intelligence (image mining) emerged as promising strategies for advanced image analysis with various purposes. Broadly, radiomics in PET images quantifies the heterogeneity of tracer uptake within a region or volume of interest (ROI or VOI). Thereafter, data on heterogeneity extracted from images are fed into statistical models or machine learning algorithms developed for clinical purposes (e.g., prognostication). Differently, artificial intelligence-based methods using labeled images as input data, autonomously identify distinctive components of the ROI/VOI, through a “learning process”, that allow the algorithm to predict a label on unseen data (Sollini et al., 2019a; Sollini et al., 2020; Sollini et al., 2019b). Preliminary data in HL supported the use of image mining to predict patients’ outcome (Ben Bouallègue et al., 2017; Milgrom et al., 2019; Lue et al., 2019; Ganeshan et al., 2017; Knogler et al., 2014). Accordingly, literature data support the concept that radiomics features differ between lesions of refractory/relapsing HL patients from those of long-term responders. However, several methodological aspects have not been elucidated yet. Firstly, lesion to choose for radiomic feature extraction has not been defined. Secondly, feature selection strategy to adopt in view of the morphological characteristics of the lymphoma lesions. Indeed, in most cases, adenopathies are ovaloid, and lesions with different size may be contemporarily present. Consequently, volume-related features may constitute confounding factors. Lastly, definitive data on predictive ability of the radiomic-based models are lacking because published data are affected by major methodological biases.

The present study aimed at developing a methodological framework for radiomics applications in lymphoma. Our primary aim was to propose a volume-related feature selection approach. Secondarily, our objective was to test whether HL lesions within a patient share a set of radiomics features (HL signature); to test this hypothesis, we evaluated radiomics intra-patient lesions’ similarity, with the final goal to inform target lesion identification for radiomics analysis. Finally, we hypothesized that the radiomics signature is able to distinguish patients with favorable vs unfavorable outcome; we tested this hypothesis by means of inter-patient similarity analysis.

## Materials and methods

### Study design and patient selection

**Fig. 1:**
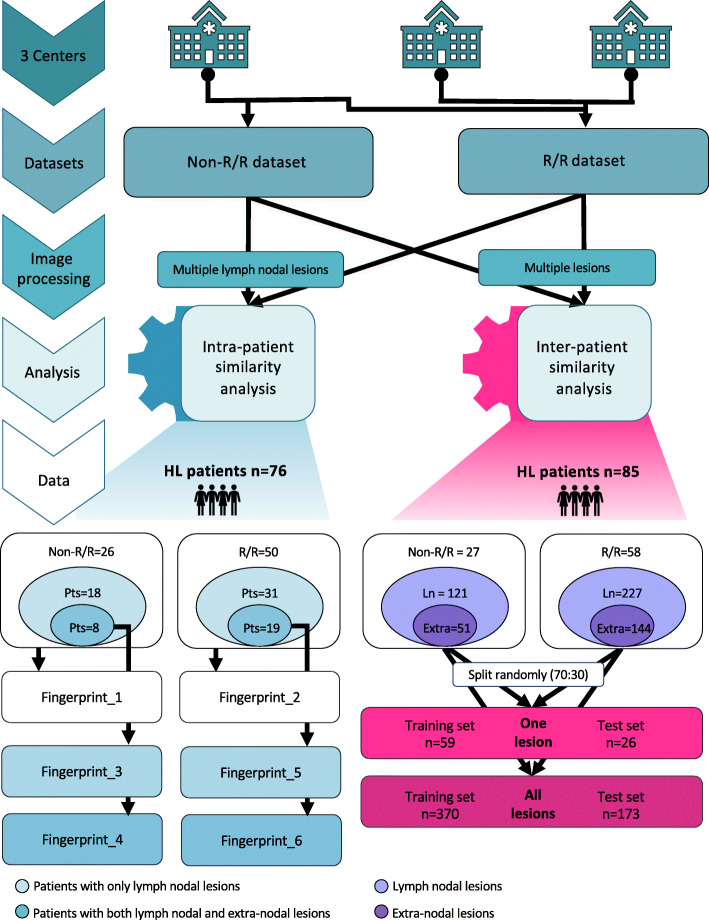
Study design

The present was an observational retrospective three-center investigation. Figure 1 shows the study design. In one center, we selected patients with pathological diagnosis of HL, who performed a pre-treatment PET/CT scan, and fell in the category of non-relapsing/refractory (i.e., long-term responder defined as disease free after at least 4 years from first-line treatment completion, non-R/R) or relapsing/refractory (R/R) treated with at least two chemotherapy lines and candidate to immunotherapy. Exclusion criteria were extravasation at injection site and no clinical data availability. In the other two centers, R/R pathologically proven HL patients candidate to immunotherapy who performed a pre-treatment PET/CT scan in loco were included. The same above-mentioned exclusion criteria were applied. Pre-treatment [^18^F]FDG-PET/CT was the baseline (i.e., staging) for non-R/R and the one before immunotherapy initiation in R/R HL patients. We identified 107 patients (male:female = 66:41; median age 35 years, range 19–74) fulfilling the inclusion/exclusion criteria. Clinical data were retrieved from the institutional records. The study, performed in accordance with the Declaration of Helsinki, was approved by the local ethics committee of all centers. The signature of a specific informed consent was waived in view of the observational retrospective study design.

### Image processing

PET/CT images were acquired according to standard institutional procedure protocols, as detailed in Supplemental Table 1. Images were retrieved and qualitatively evaluated. HL [^18^F]FDG-avid lesions were identified and classified as lymph nodal or extra-nodal, then were semi-automatically segmented with 40% of SUV_max_ threshold to define the VOI. Fifty-two radiomic features (histogram, co-occurrence and higher order, listed in Supplemental Table 2) were calculated within each VOI. The LIFEx package, version 4.9 (www.lifexsoft.org) (Nioche et al., 2018), was used for both lesion segmentation and features extraction. Lesions smaller than 64 voxels were excluded since they did not fulfil the minimum size criterion for feature extraction required by LIFEx.

## Datasets for intra-patient lesion similarity analysis

For intra-patient similarity analysis, we included patients with multiple lymph nodal lesions (> 2 lymph nodal and having or not extra-nodal lesions) of at least 64 voxels on PET images. The first dataset included non-R/R HL patients, and the second dataset included the R/R ones. Each dataset was further divided in lymph nodal and extra-nodal subsets based on lesions’ site.

## Dataset for inter-patient similarity analysis

We included patients with multiple lesions (irrespective of location:lymph nodal and/or extra-nodal) to explore the ability of the fingerprint to classify patients as non-R/R vs R/R. The classification procedure was split in a training and a test analysis using 70% and 30% of cases, respectively, preserving the composition of the original dataset.

## Statistical analysis

Patient characteristics were summarized in frequency tables, and descriptive statistics were provided. Features were normalized to Z-score prior to any model building.

## Feature selection

**Fig. 2:**
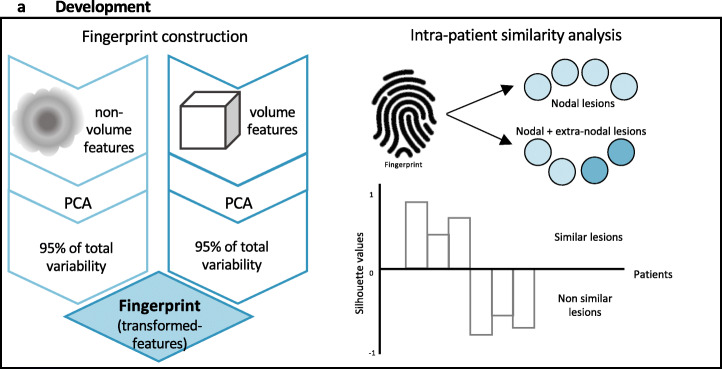
Fingerprint building and similarity analysis

For features selection, volume-related criteria were applied. In most cases, LH adenopathies are numerous. The lesions are, generally, similar being ovaloid or rounded. On the other hand, lesions with different size may be contemporarily present. Consequently, shape- and volume-related features may constitute confounding factors. Additionally, the rationale for volume-related criteria was related to the fact that in HL, typically small and large lesions co-exist, and that size affects lesion’s heterogeneity (larger lesions have been reported to be more heterogeneous than the smaller ones (Nyflot et al., 2015; Hatt et al., 2015; Sollini et al., 2017)). Therefore, the rationale for volume-related criteria was aimed at identifying all potentially relevant information and discard collinear variables, without ignoring volume component that may be relevant to predict disease aggressiveness. To do that, features were *a-priori* selected if they were highly correlated or uncorrelated to the volume (i.e., MTV) applying as cutoff a *p* value of the chi-squared test < 0.0001 as significance for uncorrelation and > 0.8 for correlation, respectively (Fig. 2).

## Fingerprint building

Selected features were then used in a principal component analysis (PCA). The transformed features accounting for at least 95% of the total variability were selected to build up the fingerprint. We built a specific fingerprint for each dataset (i.e., non-R/R and R/R) and tested the intra-patient lesions’ similarity (Fig. 2).

## Similarity analysis

The similarity index defined by the *silhouette,* computed for each patient, was used to assess intra-patient lesions’ similarity. The analyses were performed within the two groups (non-R/R and R/R), exploiting, firstly, only nodal lesions and, then, both nodal and extra-nodal lesions. Specifically, the silhouette index was computed comparing the cohesion (i.e., how similar was a lesion to other lesions of the same patient) of a lesion with its separation (i.e., how similar was a lesion to those belonging to other patients), standardizing values in order to range between − 1 and 1. Considering each patient as a grouper for the observations (i.e., lesions) belonging to him/her, one silhouette index is obtained per every patient, based on her/his lesions. Accordingly, silhouette values close to 1 indicated that the lesion well matched to those belonging to its own cluster (i.e., within the same patient) and poorly to those belonging to neighboring clusters (i.e., other patients). Vice versa, negative silhouette indicated that the lesion poorly matched to those belonging to its own cluster (i.e., within the same patient) and well to those belonging to neighboring clusters (i.e., other patients) (Fig. 2). The silhouettes computed within datasets (non-R/R and R/R) and subsets (nodal and extra-nodal) were compared. Particularly, in the nodal subset, the histogram of the silhouette values for non-R/R and R/R dataset was computed, and mean and median were compared. In the nodal + extra-nodal subset, the variation in the silhouette values was analyzed. Details on computation of the *silhouette* index are provided in the Supplementary material.

To test inter-patient similarity (i.e., classify patients as non-R/R and R/R), we used the Random Undersampling Boosting of Tree Ensemble (RUBTE)—suitable for unbalanced data (Seiffert et al., 2010). A logistic regression (LR) including only the silhouette value for each patient (based on both nodal + extra-nodal lesions) was settled in order to investigate the discrimination power of the silhouette. Classification was then performed using one lesion per patient (setting 1) and all lesions, in a data augmentation like perspective, (setting 2). Data augmentation is a typical strategy to overcome the overfitting phenomenon—a common problem related to machine learning approaches. Indeed, overfitting occurs when a high dimensional space (typical in the case of high dimensional covariates) is used for fitting data where the number of observations is not sufficiently high. Therefore, the fit of the algorithm on data is close to interpolation in the training phase. In doing so, the performances in reproducing the observed phenomenon are optimal, but then the algorithm fails in predicting unseen data during test phase, due to the insufficient ability of estimating the variability of the prediction. “Artificially” augmenting the data enables to add such variability in order to improve the performance of the algorithm in terms of prediction generality. Different strategies aimed to “artificially” augment the data may be used (van Dyk & Meng, 2001). In the setting 2, the silhouette value (equal for all the lesions belonging to the same patient) was used as a grouping factor. Thereafter, the majority vote rule was used for aggregating responses available at lesions level to a single response at patient level (i.e., for aggregating multiple lesions into a single outcome—patient R/R or patient non-R/R). Accordingly, the patient was assigned to the class R/R or non-R/R according to the majority of her/his lesion assignments (Penrose, 1946). We built a specific fingerprint per each setting (single versus all patient lesions) using the abovementioned framework for feature normalization, selection, reduction, and PCA. Conventional metrics including sensitivity, specificity, and accuracy were used to test the RUBTE performance.

## Results

### Intra-patient similarity

#### Datasets for intra-patient lesion similarity analysis

Seventy-six patients resulted to have multiple nodal lesions. Intra-patient nodal lesion similarity was tested in 26 non-R/R and 50 R/R patients.

Twenty-seven patients had both nodal and extra-nodal lesions. The intra-patient nodal + extra-nodal lesion similarity was tested in 8 non-R/R and 19 R/R cases (Table 1 and Fig. 1).

**Table 1:** Baseline characteristics of HL patients with both nodal and extra-nodal multiple lesions

	Non-R/R	R/R	Overall
Age, years			
Median and range	46 (19–66)	33 (24–71)	35 (19–71)
Sex			
Male	5	15	20
Female	3	4	7
Target HL lesions, *n*			
Nodal	48	105	153
Extra-nodal	50	134	184
Bone	46	84	130
Liver	2	5	7
Lung	–	19	19
Spleen	2	24	26
Other	–	2	2
Overall (nodal + extra-nodal)	98	239	337
Mean lesion number ± standard deviation	12 ± 9	13 ± 11	12 ± 10
Median lesion number, range	10 (4–27)	9 (3–40)	9 (3–40)

#### Fingerprints for intra-patient lesion similarity analysis

We built one fingerprint for each dataset and subset using the volume highly correlated and unrelated features, as detailed in Table 2.

**Table 2:** Fingerprints’ construction for intra-patient lesion similarity analysis

		Fingerprint_1	Fingerprint_2	Fingerprint_3	Fingerprint_4	Fingerprint_5	Fingerprint_6
**Dataset HL, type**		Non-R/R	R/R	Non-R/R		R/R	
**Patients,*n***		26	50	8		19	
**Subset**	**Lesions, site**	Nodal		Nodal	Nodal + extra-nodal	Nodal	Nodal + extra-nodal
	**Lesions,*n***	120	227	48	98	105	239
**Features volume-related**	**Name**	SUVpeak_Sphere_1mL_ TLG Volume_mL Volume_voxels Compacity Correlation_GLCM_ Entropy_log10_GLCM_ Entropy_log2_GLCM_ GLNU_GLRLM_ RLNU_GLRLM_ Coarseness_NGLDM_ Busyness_NGLDM_ LZHGE_GLZLM_ GLNU_GLZLM_ ZLNU_GLZLM_	TLG Volume_mL Volume_voxels Compacity Correlation_GLCM_ GLNU_GLRLM_ RLNU_GLRLM_ Coarseness_NGLDM_ Busyness_NGLDM_ LZE_GLZLM_ GLNU_GLZLM_ ZLNU_GLZLM_	SUVpeak_Sphere_1mL_ TLG Volume_mL Volume_voxels Compacity Entropy_log10_GLCM_ Entropy_log2_GLCM_ GLNU_GLRLM_ RLNU_GLRLM_ Coarseness_NGLDM_ Busyness _NGLDM_ GLNU_GLZLM_ ZLNU_GLZLM_	TLG Volume_mL Volume_vx Compacity GLNU_GLRLM_ RLNU_GLRLM_ Busyness_NGLDM_ GLNU_GLZLM_ ZLNU_GLZLM_	SUVpeak_Sphere_1mL_ TLG Volume_mL Volume_voxels Compacity Correlation_GLCM_ Entropy_log10_GLCM_ Entropy_log2_GLCM_ GLNU _GLRLM_ GLRLM_RLNU Coarseness_NGLDM_ Busyness_NGLDM_ GLNU_GLZLM_ ZLNU_GLZLM_	TLG Volume_mL Volume_voxels Compacity Correlation_GLCM_ GLNU_GLRLM_ RLNU_GLRLM_ Busyness_NGLDM_ LZE_GLZLM_ LZHGE_GLZLM_ GLNU_GLZLM_ ZLNU_GLZLM_
	**Number**	15	12	13	9	14	12
**Features non-volume-related**	**Name**	SUVstd Skewness_HISTO_	SUVmin SUVmean SUVmax SUVQ1 SUVQ2 Kurtosis_HISTO_ ExcessKurtosis_HISTO_ Energy_HISTO_ LRLGE_GLRLM_ LRHGE_GLRLM_	Skewness_HISTO_ LZE_GLZLM_ LZLGE_GLZLM_	SUVmin SUVQ3 HGRE_GLRLM_ SRHGE_GLRLM_ HGZE_GLZLM_ SZHGE_GLZLM_	Skewness_HISTO_ Energy_HISTO_ LZLGE_GLZLM_	LRHGE_GLRLM_ SZHGE_GLZLM_
	**Number**	2	10	3	6	3	2
**PCA retained transformed features (mapping volume + non-volume data)**		6 + 2	5 + 4	4 + 2	2 + 1	6 + 3	5 + 2

*HL* Hodgkin’s lymphoma, *n* number, *non-R/R* non-relapsing/refractory, *PCA* principal component analysis, *R/R* relapsing/refractory. For the full spelling of the feature and matrixes names, please refer to the supplementary material

Fingerprints_1 and _2 were used to explore the intra-patient nodal lesion similarity in non-R/R and R/R patients, respectively. Fingerprints_3 and _4 were built on nodal and nodal + extra-nodal non-R/R lesions, respectively. Fingerprints_5 and _6 were built on nodal and nodal + extra-nodal R/R lesions, respectively (Fig. 1).

#### Intra-patient nodal lesion similarity analysis

**Fig. 3:**
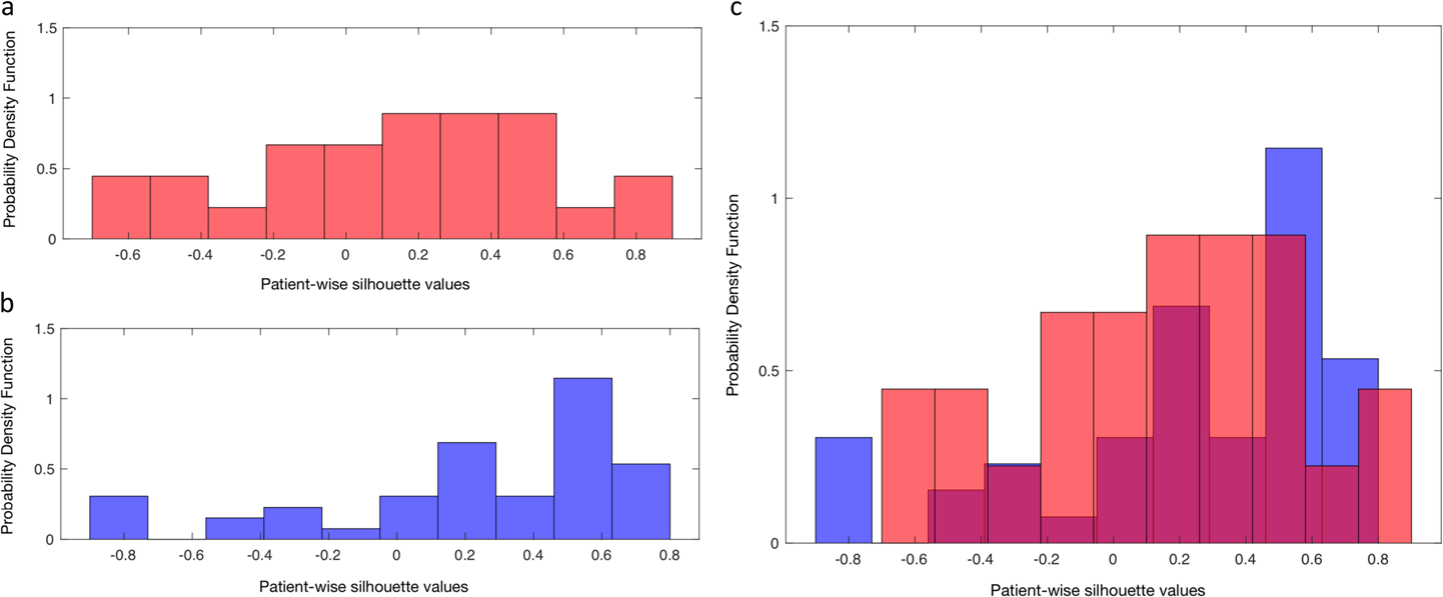
Histograms of the silhouette values. Histogram of the silhouette values of lymph nodal lesions in non-relapsing/refractory **(a**) and relapsing/refractory (**b**) patients. The overlap of two histograms (**c**) shows a more uniform distribution of the silhouettes in the non-relapsing/refractory compared to the relapsing/refractory ones

In the nodal non-R/R subset, 18/26 (69%) silhouette values resulted positive using fingerprint_1 (mean 0.11 ± 0.42). The histogram of the silhouette values is shown in Figure 3a.

In the nodal R/R subset, 38/50 (76%) silhouettes resulted positive using fingerprint_2 (mean 0.24 ± 0.45). The histogram of such values is given in Fig. 3b. Figure 3c shows the overlap of the two histograms. Overall, the silhouettes in the non-R/R dataset showed a more uniform distribution compared to the R/R ones.

The mean values of the distributions of the silhouettes in non-R/R and R/R resulted not statistically different (*p* value = 0.08). Conversely, the median value of non-R/R was lower than the median value of R/R (0.11 versus 0.39). Overall, the comparison between histograms demonstrated a higher intra-patient lesion similarity in the R/R dataset than in the non-R/R one.

#### Intra-patient nodal and extra-nodal lesion similarity analysis

**Fig. 4:**
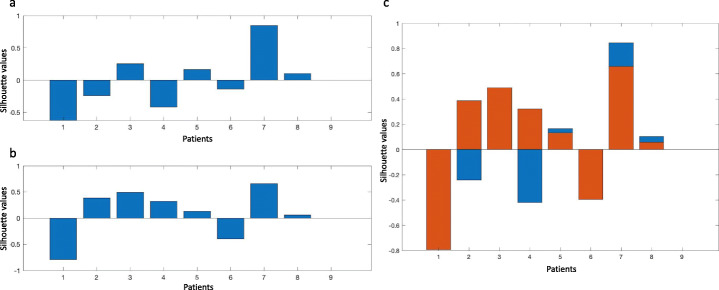
Silhouette results among non-relapsing/refractory patients. Patient-wise silhouette among non-relapsing/refractory patients with lymph nodal (**a**) and all (**b**) lesions. Variations of patient-wise silhouette with/without extra-nodal lesions (**c**)

In the non-R/R dataset, only 4/8 (50%) silhouettes resulted positive using the fingerptint_3 (mean -0.01 ± 0.46), as shown in Figure 4a. If both lymph nodal and extra-nodal lesions were considered, 6/8 (75%) silhouettes had positive values (mean 0.12 ± 0.61, Figure 4b).

**Fig. 5:**
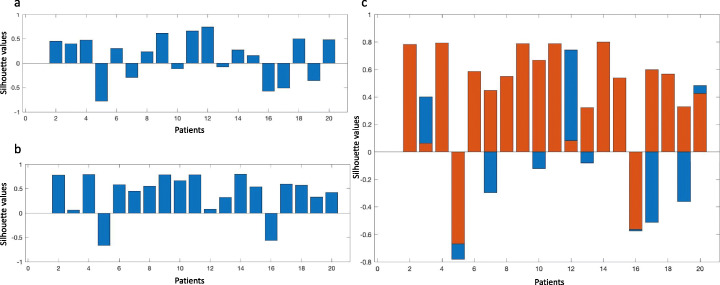
Silhouette results among relapsing/refractory patients. Patient-wise silhouette among relapsing/refractory patients with lymph nodal (**a**) and all (**b**) lesions. Variations of patient-wise silhouette with/without extra-nodal lesions (**c**)

In the R/R dataset, 12/19 (63%) silhouettes resulted positive using the fingerptint_5 (mean 0.13 ± 0.46, Fig. 5a). Seventeen out of nineteen (90%) silhouettes had positive values (mean 0.42 ± 0.43) when both nodal and extra-nodal lesions were used (Fig. 5b).

The introduction of extra-nodal lesions improved the silhouette index in 3 and in 5 patients in non-R/R (Fig. 4c) and R/R datasets (Fig. 5c), respectively. Overall, these results demonstrated a higher intra-patient lesion similarity in the R/R dataset than in the non-R/R.

### Inter-patient similarity

#### Datasets for inter-patient similarity analysis

Eighty-five patients resulted to have multiple lesions (Table 3 and Fig. 1). Eighteen out of 27 non-R/R patients had only lymph nodal lesions, while 9/27 patients had both lymph nodal and extra-nodal lesions.

**Table 3:** Baseline characteristics of HL patients with multiple lesions

	Non-R/R	R/R	Overall
Age, years			
Median and range	42 (19–66)	33 (19–74)	35 (19–74)
Sex			
Male	15	37	52
Female	12	21	33
Target HL lesions, *n*			
Nodal	121	227	348
Extra-nodal	51	144	195
Bone	47	89	136
Liver	2	5	7
Lung	–	24	24
Spleen	2	24	26
Other	–	2	2
Overall (nodal + extra-nodal)	172	371	543
Mean lesions number ± standard deviation	6 (± 6)	6 (± 8)	6 (± 7)
Median lesions number, range	4 (2–27)	4 (2–40)	4 (2–40)

In the R/R dataset, 36/58 patients had only lymph nodal lesions, 20/58 patients had both lymph nodal and extra-nodal lesions, and 2/58 patients had only extra-nodal lesions.

When one lesion was used for the classification (setting 1), the training set included 22 non-R/R and 37 R/R lesions, while the test set included 9 non-R/R and 17 R/R lesions.

When all lesions were used for the classification (setting 2), the training set included 115 non-R/R and 255 R/R lesions, respectively. The test set included 57 non-R/R and 116 R/R lesions, respectively.

#### Fingerprints for inter-patient similarity analysis

Intra-patient lesion similarity analysis results, with mean/median silhouette values below 0.5 (low similarity especially in the non-R/R group), did not support the random choice of a target lesion for inter-patient similarity analysis. Therefore, two alternative approaches were tested for classification. Firstly, the largest nodal or extra-nodal lesion, as for conventional approach, was used for the classification (fingerprint_One). The fingerprint_All was built using all nodal and extra-nodal lesions. Details about fingerprints built for inter-patient similarity analysis, including the volume highly correlated and unrelated features, are provided in Table 4.

**Table 4:** Fingerprints construction for inter-patient similarity analysis

		Fingerprint_One	Fingerprint_All
**Dataset HL, type**		Non-R/R + R/R	Non-R/R + R/R
**Patients,*n***		85	85
**Subset**	**Lesions, site**	Nodal or extra-nodal	Nodal + extra-nodal
	**Lesions,*n***	85	543
**Features volume-related**	**Name**	TLG Volume_mL Volume_vx Compacity GLNU_GLRLM_ RLNU_GLRLM_ GLNU_GLZLM_ ZLNU_GLZLM_	TLG Volume_mL Volume_vx Compacity Correlation_GLCM_ GLNU_GLRLM_ RLNU_GLRLM_ Coarseness _NGLDM_ Busyness _NGLDM_ LZE_GLZLM_ LZHGE_GLZLM_ GLNU_GLZLM_ ZLNU_GLZLM_ ZP_GLZLM_
	**Number**	8	14
**Features non-volume-related**	**Name**	SUVmean SUVQ2 SUVQ3 Entropy_log10 _HISTO_ Entropy_log2 _HISTO_ Energy_HISTO_ LGRE_GLRLM_ HGRE_GLRLM_ SRHGE_GLRLM_ LRLGE_GLRLM_ LRHGE_GLRLM_ HGZE _LZLM_	SUVQ3 Entropy_log10 _HISTO_ Entropy_log2 _HISTO_ HGRE_GLRLM_ SRHGE_GLRLM_ LRHGE_GLRLM_ HGZE_GLZLM_
	**Number**	12	7
**PCA retained transformed features (mapping volume + non-volume data)**		2 + 2	7 + 2

*HL* Hodgkin’s lymphoma, *n* number, *non-R/R* non-relapsing/refractory, *PCA* principal component analysis, *R/R* relapsing/refractory. For the full spelling of the feature and matrixes names, please refer to the supplementary material

#### Inter-patient similarity analysis

The classification accuracy based on fingerprint_One was 62% with 78% of sensitivity and 53% of specificity in the test set.

The silhouette value significantly discriminated non-R/R from R/R (odds ratio = 1.85). Therefore, it was included in the RUBTE as grouping factor for lesions belonging to the same patient. The RUBTE sensitivity and specificity in the test set were 70% and 88%, respectively (accuracy = 82%).

When lesions were aggregated at patient’s level through the “majority vote,” the ability in discriminating R/R raised to 89% (accuracy = 73%), but a significant loss in sensitivity was observed (38% versus 88%).

## Discussion

We proposed a volume-related feature selection approach to reduce dimensionality and identify those parameters that are relevant for HL characterization. It is known that a multitude of radiomics parameters are strongly correlated one to another, implying high redundancy that affects radiomics models’ performance. Dimensionality reduction and feature selection are crucial mandatory steps before any modeling (Sollini et al., 2020; Park et al., 2019). In fact, as recommended, an adequate ratio between the number of features and the number of patients should be preserved. Additionally, lesion morphology and size in lymphoma patients are similar within and among patients. Therefore, radiomics features unbiased from the volume and shape descriptors need to be identified in radiomics applications in lymphoma. Our approach allowed us to select a set of features (ranging from 2 to 15) to be used for model building. The main advantage of the proposed method for features reduction and selection relies on the concept that a fingerprint, comprising volume-related and non-volume related features, is able to represent all the lesions of a patient.

In view of the multisite disease, identification of the lymphoma lesion to be processed for radiomics analyses is crucial. We proposed an innovative approach for radiomic-wise lesion similarity assessment to provide the evidence for target selection. Conventionally, the largest lesion or the one with the highest FDG uptake is used but the rationale for this method has never been supported by any evidence. We demonstrated that the lesions within a patient may show different grades of similarity. Intra-patient lesion similarity within R/R patients was higher compared to non-R/R. Interestingly, intra-patient lesion similarity in the R/R dataset was confirmed also when extra-nodal lesions were included in the analysis. In the non-R/R group, the addition of extra-nodal lesions to nodal ones had a minor effect on similarity.

The non-relapsing/refractory (non-R/R) group is a homogeneous subset of patients; it included all cases before treatment initiation, and they were included in one single institution. On the other hand, the relapsing/refractory (R/R) group included patients treated with several lines of treatment coming from different centers. These two scenarios allowed us to explore lesion similarity in two opposite situations. Furthermore, we aimed at identifying a radiomic fingerprint that could be representative of HL lesions irrespective of all the variables, with the long-term goal of wide application of the fingerprint among different centers. We, indeed, found that the R/R, even if it could be expected to be more heterogeneous, resulted to have higher intra-patient similarity as compared to non-R/R.

It should be acknowledged that the number of observations (i.e., lesions) may have partially affected these findings. However, we did not expect to provide definitive results but to propose a methodological framework for future investigations. Indeed, our “proof-of-concept” approach resulted encouraging for further development for response prediction. We foresee the necessity of research in this direction since among the available studies, the bias related to a significant disproportion between the patient groups (responders vs non-responders being the latter less than 10% of the whole cohort (Milgrom et al., 2019)) may have significantly influenced the results. Additionally, the intra-patient lesion similarity in non-R/R patients was scarce even when a higher number of lesions were analyzed (Fig. 3a), suggesting that this group of patients was intrinsically more heterogenous. This finding was expected since non-R/R HL, naïve from any treatment, included patients who later on experienced long-term response, relapse, and refractory disease; therefore, it was the most heterogenous group. Conversely, R/R patients may be biologically more homogeneous, since treatments might result in resistant clones’ selection. Moreover, the non-neoplastic cells of tumor microenvironment have been claimed as one of the main determinants responsible for pathogenesis and progression of HL (Mottok & Steidl, 2018; Calabretta et al., 2019). Infiltration of the tumor microenvironment by CD68+ and CD163+ macrophages, Treg and CD4^+^ T cells (especially with Th2 phenotype), and high CD4/CD8 ratio is associated to the emergence of resistance to conventional therapy, and a worse prognosis. Additional factors that dysregulate tumor microenvironment promote a vicious loop between malignant cells and the components of the tumormicroenvironment stimulating resistance to treatment and disease progression. These factors include the recruitment of tumor-associated macrophages, the secretion of cytokines with macrophage chemotactic activity reinforced by the reactive cells, the activation of fibroblasts promoted by molecules secreted by malignant cells, the expression of surface antigens (e.g., CD30L, CD40L) by inflammatory cells that act as survival signals for the neoplastic cells, and the aberrant activation of signaling pathways (e.g., NF-κB, PI3K) (Karantanos et al., 2017) promote a vicious loop between malignant cells and the components of the tumor microenvironment stimulating resistance to treatment and disease progression (Karantanos et al., 2017). Of note, evidence suggests that [^18^F]FDG uptake is more likely related to elements of microenvironment rather than malignant HL cells (Gillessen et al., 2020; Barrington & Mikhaeel, 2014; Shim et al., 2009). Accordingly, our findings are in line with the fact that heterogeneity of the tumor microenvironment in naïve patients is more pronounced than that of R/R patients. Therefore, our results support the need for development of a radiomics fingerprint in a large cohort of naïve patients. Essentially, in this analysis, we explored and developed a framework for radiomics analysis in lymphoma. Simultaneous presence of many lesions is a typical finding in lymphoma, and recent data on molecular profiles suggest lesions’ heterogeneity (Spina et al., 2018; Banerjee, 2011).

The question, related to the choice of which and/or how many lesions, which guide the disease, and need to be processed, is unresolved. In image mining studies, one possible approach to address this issue is the choice of the largest and/or the most metabolically active lesion, as for conventional image analysis and adopted by previous studies (Ben Bouallègue et al., 2017; Tatsumi et al., 2019). However, large heterogeneous lesions (often necrotic or with multiple uptake peaks) may underestimate the volume (El-Galaly et al., 2018; Carles et al., 2017) and influence texture measurements. On the other hand, all the lesions could be considered for radiomics analysis. As demonstrated in the present study, enriching the analysis through the use of the information derived from all lesions improved the classification performance. Results of the classifier using the largest lesion were not satisfactory (accuracy = 60%), but the small sample size prevents any speculation about their reliability. Conversely, the RUBTE provided promising results when all lesions were used for the analysis, similarly to the previous investigations (Lue et al., 2019; Ganeshan et al., 2017; Parvez et al., 2018; Mayerhoefer et al., 2019). Unlike in the study by Milgrom et al., the authors found the mediastinal lesion-derived features could predict patient outcome, while features extracted from all lymphoma sites did not predict refractory disease (Milgrom et al., 2019). Overall, segmentation or annotation of all lesions is time-consuming and could hardly be implemented into the clinical routine practice. Therefore, suitable trade-off considering the number of cases at hand and the needed predictive power is necessary.

HL typically involves more than one site, and lesions different in size may co-exist. We found that the PCA-derived information mapping volume data outnumbered non-volume ones in almost all cases, with the exception of fingerprint_One—the one built on the largest lesion. Therefore, the huge variability in lesions’ size within patients required more covariates (i.e., features) to characterize the lesions and to be inclusive for all lesions. Our results are encouraging for exploring the proposed framework in larger multicenter trials. We foresee a replication study to confirm our data. Secondly, we propose that future radiomics investigations on lymphoma have to rely on the radiomics features derived from all the lesions of a patient. The approach we developed may be applied also for solid tumor studies if multiple lesions are present, in order to understand from which lesion (primary, secondary or all) to extract the features for modeling and predictions.

Some limitations should be acknowledged including the retrospective design and sample size, even if the involvement of more centers conferred strength to results. We pooled features extracted from images acquired using different scanners (Orlhac et al., 2018). On the other hand, we did not search for feature cutoff in the analysis. Moreover, we had previously demonstrated that scanners and image postprocessing did not affect final results (Kirienko et al., 2018). Additionally, to test our research hypothesis, we evaluated the lesions within the same patient; therefore, the scanning protocol and postprocessing were consistent among lesions. We developed a fingerprint for each group of patients. Obviously, the development of one fingerprint representative for all lesions regardless the site (nodal or extra-nodal) and the dataset (non-R/R or R/R) would be the ultimate goal. However, the primary aim of this preliminary analysis was to test if really radiomics differed in non-R/R and R/R (i.e., define a methodological framework to demonstrate the potential predictive value of radiomics in HL). Background activity may affect segmentation and, consequently, feature calculation. Nonetheless, the introduction of extra-nodal lesions improved the silhouette index in non-R/R (Fig. 4c) and R/R datasets (Fig. 5c), respectively. We could speculate that, irrespective of the possible issues in extra-nodal lesions segmentation, lesion texture did not result in higher inhomogeneity. However, these results should be confirmed in larger datasets, since in our cohort only 27 patients had extra-nodal lesions. When lesions were adjacent to areas of high physiological uptake, we avoided to include those lesions for radiomics analysis in order not to introduce a bias in lesion segmentation. We operated that choice since we expected it to be more robust and generalizable for future studies. Additionally, we decided to avoid considering diffuse uptake disease in bone, spleen, and liver in the present analysis in order not to introduce a potential bias in image interpretation since diffuse uptake may have been related to both disease infiltration and functional activation. Lastly, within the inter-patient analysis, we compared patient populations in two different settings—naïve patients at staging (non-R/R) and patients candidate to immunotherapy who failed several lines of treatment (R/R). This choice was based on the expectation that the class of non-R/R HL accounting for patients that did not recurred after at least 4 years from first-line treatment completion (i.e., cured HL) would have differed the most from the class of R/R.

## Conclusions

We proposed a novel approach for radiomics feature selection that allowed to build patient representative radiomics signatures. Lesion similarity analysis was developed, and it allowed to demonstrate that HL lesions were not homogeneous within the patients in terms of radiomics signature. Therefore, a random target lesion selection should not be adopted for radiomics applications. Moreover, the classifier to predict R/R vs non-R/R performed the best when all the lesions were used. This implies that the largest lesion is not reliable, and that the information coming from different lesions contribute to patient outcome prediction.

## Supplementary information

**Additional file 1: Supplementary Table S1.** PET/CT images acquisition parameters.

**Additional file 2: Supplementary Table 2.** Radiomics features calculation report according to the Imaging Biomarkers Standardization Initiative (IBSI) manual. Detailed description of silhouette computation.

## Data Availability

The datasets used and/or analyzed during the current study are available from the corresponding author on reasonable request.

## Abbreviations

[^18^F]FDG: 2-Deoxy-2-[^18^F]fluoroglucose
PCA: Principal discriminant analysis
ICA: Independent discriminant analysis
n.a.: Not applicable
ROI: Region of interest
VOI: Volume of interest
MTV: Metabolic tumor volume
SUV: Standardized uptake value
HL: Hodgkin’s lymphoma
Non-R/R: Non-refractory/relapsing
R/R: Refractory/relapsing
