# Supplementary material for: Methodological framework for radiomics applications in Hodgkin’s lymphoma
Source: Eur J Hybrid Imaging. 2020 Jun 1;4:9. doi: 10.1186/s41824-020-00078-8 (PMC8218114; doi:10.1186/s41824-020-00078-8)
Supplement: Supplementary file 1 — Additional file 1: Supplementary Table S1. PET/CT images acquisition parameters. [file 41824_2020_78_MOESM1_ESM.docx]

**Supplemental Table 1**. Image acquisition protocols.

| **Humanitas Clinical and Research Center** | | | | | **Fondazione IRCCS Istituto Nazionale dei Tumori** | | | | **AOU S.Orsola-Malpighi Bologna** | |
| --- | --- | --- | --- | --- | --- | --- | --- | --- | --- | --- |
| **Acquisition parameters** | **Biograph – Siemens** | | **Discovery 690 – GE** | | **Discovery 710 – GE** | | **Gemini - Philips** | | **Discovery STE - GE** | |
|  | PET | CT | PET | CT | PET | CT | PET | CT | PET | CT |
| **Min/bed position**  **(static/dynamic)** | 2.5 (static) | – | 2 (static) | – | 2 (static) | – | 2 (static) | – | 2 (static) | - |
| **Crystal** | LSO | – | LYSO | – | LYSO | – | BGO | – | LBS | – |
| **Reconstruction** | Iterative | – | Iterative, TOF Sharp IR | – | VPFX | – | Iterative | – | Iterative OSEM, PFS | – |
| **Attenuation correction** | On CT data | – | On CT data | – | On CT data | – | On CT data | – | On CT data | – |
| **Matrix (pixels)** | 128×128 | 512×512 | 256×256 | 512×512 | 192×192 | 512×512 | 169×169 | 512×512 | 256×256 | 512×512 |
| **Resolution (mm)** | 5.3×5.3 | 0.98×0.98 | 2.73×2.73 | 1.37×1.37 | 3.65×3.65 | 1.37×1.37 | 4×4 | 1.37×1.37 | 4x4 | 0.625x0.625 |
| **Slice thickness (mm)** | 2.0 | 4.0 | 3.27 | 3.27 | 3.27 | 3.75 | 4 | 4 | 3.27 | 3.27 |
| **Slices** | – | 6 | – | 64 | – | 64 | – | 64 | – | 16 |
| **Voltage (kV)** | – | 130 | – | 140 | – | 120 | – | 120 | – | 120 |
| **Tube current (mA)** | – | 95 | – | 140 | – | 40-120 | – | 60-120 | – | 80 |
