# Supplementary material for: Methodological framework for radiomics applications in Hodgkin’s lymphoma
Source: Eur J Hybrid Imaging. 2020 Jun 1;4:9. doi: 10.1186/s41824-020-00078-8 (PMC8218114; doi:10.1186/s41824-020-00078-8)
Supplement: Supplementary file 2 — Additional file 2: Supplementary Table 2. Radiomics features calculation report according to the Imaging Biomarkers Standardization Initiative (IBSI) manual. Detailed description of silhouette computation. [file 41824_2020_78_MOESM2_ESM.docx]

**Supplemental Table 2.** Radiomics features calculation report according to the Imaging Biomarkers Standardization Initiative (IBSI) manual v.8 [1].

| **General** | |
| --- | --- |
| Imaging | Total-body (from the skull base to the mid-thigh) positron emission tomography (PET); standard protocol according to EANM procedure guidelines for tumor imaging version 2.0 [2] |
| Patient preparation | Fasting for at least 4 hours before injection; blood glucose levels below 200 mg/dl were requested |
| Radioactive tracer | ^18^F-fluorodeoxyglucose ([^18^F]FDG); Intravenous administration  Injected activity range (185-550 MBq)  Uptake time range 55-65 minutes |
| Acquisition | See suppl. Table 1 |
| Approach | The images were analysed as a volume (3D) |
| Process structure | Image acquisition -> reconstruction -> segmentation -> export -> texture analysis -> feature calculation report |
| Software | LIFEx 4.9, (www.lifexsoft.org) [3] |
| Data availability | All the original patient anonymised DICOM files are stored on the department hard disk. The calculations of the features are stored in the department hard disk. |
| **Data conversion** | |
| Procedure | None |
| **Image post-acquisition processing** | |
| Procedure | None |
| **Segmentation** | |
| ROI | The volume of interest (VOI) included the primary tumour |
| Procedure | The ROIs were semi-automatically defined on PET images (with 40% of SUV_max_ threshold) using LIFEx [3]  When lesions were adjacent to areas of high physiological uptake, we avoided to include those lesions for radiomics analysis in order not to introduce a bias in lesion segmentation.  In case of two adjacent lesions, the following approach was used. In case of lesions close to each other, if the lesions could be distinguished, manual segmentation was applied, and final segmentation was reached by consensus with another experienced nuclear medicine physician. If the lesions could not be clearly separated, the lesion was considered as a single one and semi-automatic delineation with a threshold of 40% of the SUVmax was used. |
| **Interpolation** | Not performed |
| Voxel dimensions | Not applicable |
| Image interpolation method | Not applicable |
| Image intensity rounding | Not applicable |
| ROI interpolation method | Not applicable |
| ROI partial volume | Not applicable |
| **Re-segmentation** |  |
| ROI mask criteria | Not applicable |
| **Discretisation** |  |
| Discretisation method | 64 bins, absolute intensity rescaling between 0 and 20 SUV units |
| **Feature calculation** |  |
| Feature set | **Conventional**  *SUVmin* (reflects the minimum Standardized Uptake Value (SUV) in the Volume of Interest)  *SUVmean* (reflects the average Standardized Uptake Value (SUV) in the Volume of Interest)  SUVstd (reflects the standard deviation of Standardized Uptake Value (SUV) in the Volume of Interest)  *SUVmax* (reflects the maximum Standardized Uptake Value (SUV) in the Volume of Interest)  *SUVpeak* (reflects the mean SUV in a sphere with a volume of ~0.5 or ~1 mL and located so that the average value in the VOI is maximum)  *TLG* (is the Total Lesion Glycolysis defined as the product of SUVmeanSUVmean by VolumeVolume in mL)  **Histogram-based**  *Skewness_HISTO_* (is the asymmetry of the grey-level distribution in the histogram)  *Kurtosis_HISTO_*(reflects the shape of the grey-level distribution (peaked or flat) relative to a normal distribution)  *Entropy_HISTO_*(reflects the randomness of the distribution)  *Energy_HISTO_*(reflects the uniformity of the distribution)  **Geometry-based**  *Volume*  *Sphericity* (is how spherical a Volume of Interest is)  *Compacity*  **Grey level co-occurrence (takes into account the arrangements of pairs of voxels to calculate textural indices)**  *Homogeneity* (the homogeneity of grey-level voxel pairs)  *Energy_GLCM_* (the uniformity of grey-level voxel pairs)  *Contrast_GLCM_* (the local variations in the GLCM)  *Correlation* (the linear dependency of grey-levels in GLCM)  *Entropy_GLCM_* (the randomness of grey-level voxel pairs)  *Dissimilarity* (the variation of grey-level voxel pairs)  *Entropy_log10_GLCM_* (the randomness of grey-level voxel pairs)  *Entropy_log2_GLCM_* (the randomness of grey-level voxel pairs)  **Neighbourhood grey level difference matrix (corresponds to the difference of grey-level between one voxel and its 26 neighbours in 3 dimensions)**  *Contrast* (the intensity difference between neighbouring regions)  *Coarseness* (the level of spatial rate of change in intensity)  **Grey level run length matrix (gives the size of homogeneous runs for each grey level)**  *Short-run emphasis* (SRE)(the distribution of the short homogeneous runs in an image)  *Long-run emphasis* (LRE) (the distribution of the long homogeneous runs in an image)  *Low grey level run emphasis* (LGRE)(is the distribution of the low grey-level runs)  *High grey level run emphasis* (HGRE)(is the distribution of the high grey-level runs)  *Short-run low grey level emphasis* (SRLGE)(the distribution of the short homogeneous runs with low grey-levels)  *Short-run high grey level emphasis* (SRHGE)(the distribution of the short homogeneous runs with high grey-levels)  *Long-run low grey level emphasis* (LRLGE)(the distribution of the long homogeneous runs with low grey-levels)  *Long-run high grey level emphasis* (LRHGE) (the distribution of the long homogeneous runs with high grey-levels)  *Grey level non-uniformity for run* (GLNU_GLRLM_) (the non-uniformity of the grey-levels)  *Run length non-uniformity* (RLNU_GLRLM_)(the length of the homogeneous runs)  *Run percentage* (RP)(measures the homogeneity of the homogeneous runs)  **Grey level zone length matrix (provides information on the size of homogeneous zones for each grey-level in 3 dimensions)**  *Short-zone emphasis* (SZE) (distribution of the short homogeneous zones in an image)  *Long-zone emphasis* (LZE) (distribution of the long homogeneous zones in an image)  *Low grey level zone emphasis* (LGLZE) (is the distribution of the low grey-level zones)  *High grey level zone emphasis* (HGLZE) (is the distribution of the high grey-level zones)  *Short-zone low grey level emphasis* (SZLGLE)(the distribution of the short homogeneous zones with low grey-levels)  *Short-zone high grey level emphasis* (SZHGLE)(the distribution of the short homogeneous zones with high grey-levels)  *Long-zone low grey level emphasis* (LZLGLE)(the distribution of the long homogeneous zones with low grey-levels)  *Long-zone high grey level emphasis* (LZHGLE)(the distribution of the long homogeneous zones with high grey-levels)  *Grey level non-uniformity for zone* (GLNU_GLZLM_) (the non-uniformity of the grey-levels)  *Zone length non-uniformity* (ZLNU)(the length of the homogeneous zones)  *Zone percentage* (ZP) (measures the homogeneity of the homogeneous zones) |
| Feature parameters | The parameters were calculated according to the formulas described in the LIFEx 4.9 manual [3] |
| **Standardisation** | Not performed |

**Detailed description of *silhouette* computation**

The silhouette value provides a measure of how similar an object (lesion) is to its own cluster (patient), i.e. cohesion, compared to other clusters (patients), i.e. separation. The silhouette ranges from −1 to +1, where a high value indicates that the object is well matched to its own cluster and poorly matched to neighboring clusters. If most objects have a high value, then the similarity assumption is appropriate. If many points have a low or negative value, then the clustering configuration may be lacking.

The silhouette was calculated according to the Euclidean distance definition, computed as Manhattan distance with n=2 (Eq 1). Specifically, the silhouette value $s(i)$ for each patient $P_{i}$ with respect to all the other patients $P_{k}$ is given by:

Equation 1.

$$s\left( P_{i} \right)= \frac{b\left( P_{i} \right)-a(P_{i})}{max\{a(P_{i}), b(P_{i})\}}, if \left| P_{i} \right|>1$$

where:

$$a\left( P_{i} \right)= \frac{1}{\left| P_{i} \right|-1}\sum_{i,j\in P_{i},i\neq j} d\left( i,j \right)$$

$$b\left( P_{i} \right)=\min_{k\neq i} \frac{1}{\left| P_{k} \right|}\sum_{i\in P_{i,}k\in P_{k}} d\left( i,k \right)$$

Namely, $a(P_{i})$ and $b(P_{i})$ are respectively the mean intra-patient pairwise distance between lesions (cohesion) and minimum inter-patient pairwise distance between lesions (separation).

Practically, every lesion which meets the inclusion criteria are considered and $a(P_{i})$ and $b(P_{i})$ are computed: $a(P_{i})$ is defined as the mean distance between every lesion $i$ belonging to $P_{i}$ patient and all other lesions $j$ of the same patient, where $d(i,j)$ is their pairwise distance: we divide by ${|P}_{i}|-1$ since we might not want to include the distance $d(i,i)$ in the sum. We can interpret $a(P_{i})$ as a measure of how close to each other the lesions are in the patient (the smaller the value, the better the assignment). Similarly, $b(P_{i})$ describes the minimum distance of each lesion $i$ belonging to $P_{i}$ patient with respect to those lesions $k$ belonging to any other patient $P_{k}$, of which $i$ is not a member. $b(P_{i})$ may be explained as the closeness of one patient’s lesions to other patients’ ones, which thus result to be more or less similar.

Defined as the properly weighted comparison between cohesion and separation metrics, $s(P_{i})$ is a measure of how tightly grouped all the lesions in the patient $P_{i}$ are, that is how far is the considered patient from all the others $P_{k}$ in terms of lesion description. We thus define this metrics to be the intra-patient similarity index, eventually evaluated for every patient in the dataset. Values bigger than 0.8 were assumed as robust and appropriate to enhance the similarity assumption.

**Bibliography**

1. Zwanenburg A, Leger S, Vallières M, Löck S. Image biomarker standardisation initiative [Internet]. [cited 2019 Oct 23]. Available from: http://arxiv.org/abs/1612.07003

2. Boellaard R, Delgado-Bolton R, Oyen WJG, Giammarile F, Tatsch K, Eschner W, et al. FDG PET/CT: EANM procedure guidelines for tumour imaging: version 2.0. Eur J Nucl Med Mol Imaging. 2014;42:328–54.

3. Nioche C, Orlhac F, Boughdad S, Reuze S, Goya-Outi J, Robert C, et al. Lifex: A freeware for radiomic feature calculation in multimodality imaging to accelerate advances in the characterization of tumor heterogeneity. Cancer Res. 2018;78:4786–9.
